# Tofacitinib Treatment in Primary Herpes Simplex Encephalitis Interferes With Antiviral Response

**DOI:** 10.1093/infdis/jiac040

**Published:** 2022-02-26

**Authors:** Malgorzata Krzyzowska, Anders Jarneborn, Karolina Thorn, Kristina Eriksson, Tao Jin

**Affiliations:** Department of Rheumatology and Inflammation Research, Sahlgrenska Academy, University of Gothenburg, Gothenburg, Sweden; Military Institute of Hygiene and Epidemiology, Warsaw, Poland; Department of Rheumatology and Inflammation Research, Sahlgrenska Academy, University of Gothenburg, Gothenburg, Sweden; Department of Rheumatology, Sahlgrenska University Hospital, Gothenburg, Sweden; Department of Rheumatology and Inflammation Research, Sahlgrenska Academy, University of Gothenburg, Gothenburg, Sweden; Department of Rheumatology and Inflammation Research, Sahlgrenska Academy, University of Gothenburg, Gothenburg, Sweden; Department of Rheumatology and Inflammation Research, Sahlgrenska Academy, University of Gothenburg, Gothenburg, Sweden; Department of Rheumatology, Sahlgrenska University Hospital, Gothenburg, Sweden

**Keywords:** herpes simplex virus, microglia, monocytes, tofacitinib

## Abstract

Tofacitinib, a Janus kinase inhibitor, is a novel immunosuppressive drug for treatment of rheumatoid arthritis. Herpes simplex virus type 1 (HSV-1) may cause encephalitis during primary infection or following reactivation from a latent state. Long-term tofacitinib treatment may increase the risk of this life-threatening condition. The aim of this study was to investigate the effect of tofacitinib on HSV-1 primary infection using a mouse model. Mice pretreated with tofacitinib were intranasally infected with a clinical strain of HSV-1 and monitored for infection severity and antiviral response. Tofacitinib treatment of HSV-1 primary infection resulted in increased viral loads and worsened clinical outcome. Furthermore, tofacitinib promoted M2 anti-inflammatory phenotype of microglia and infiltrating monocytes, as well as inhibited production of inflammatory and antiviral cytokines by macrophages in vitro. Our findings show that treatment with tofacitinib increases severity of herpes simplex encephalitis in mice, by impairing antiviral response induced by monocytes and microglia.

## Teaser

This article provides novel evidence that treatment with tofacitinib poses an elevated risk of disease aggravation and development of severe encephalitis in primary herpes simplex infection. The responsible mechanism includes impairment of antiviral response induced by monocytes and microglia.

Herpes simplex virus type 1 (HSV-1) is very common in humans, with an estimated 70% of the world adult population having been exposed [1]. It causes infection in the form of oral lesions and after primary infection, latency is established in sensory neurons with a risk of recurring disease [2]. Herpes simplex encephalitis (HSE) is a rare but very serious infection, with an incidence of 2–4 per 1 million [3]. HSE is associated with a mortality of around 10% with antiviral treatment and up to 70% if untreated [4]. Survivors have a high incidence of sequelae, for example, epilepsy and neuropsychiatric conditions [3]. Neonates and elderly are at higher risk of HSE.

Janus kinase inhibitors (JAKi) are a group of small, synthetic immunomodulatory drugs for a variety of inflammatory and hematological disorders [5]. Janus kinases (JAKs) are kinases associated with the intracellular part of the receptors of a large number of cytokines and hormones. Activation of JAKs upon cytokine stimulation results in recruitment and phosphorylation of signal transducer and activator (STAT) proteins, which dimerize and translocate to the nucleus to affect transcription of a great variety of genes. There are 4 types of JAKs: JAK 1–3 and TYK2 [6]. Tofacitinib is the first JAK inhibitor to be approved for treatment of rheumatoid arthritis, and is now also used for treatment of psoriatic arthritis, ulcerative colitis, and some immune-mediated skin disorders [7, 8]. Tofacitinib blocks JAK1 and JAK3, and to a lesser extent JAK2. The JAK-STAT system is used in the signaling of a considerable number of cytokines; among the ones using primarily JAK1 and/or JAK3 are interferons (IFNs), interleukin 2 (IL-2), IL-4, and IL-9 [9]. It is known that tofacitinib increases the risk for certain infections, which is comparable to biological immunosuppressive agents such as tumor necrosis factor-α (TNF-α) [10]. One risk that stands out for tofacitinib and other JAKi is a clear increased risk for herpes zoster virus [10–12]. However, there are very limited data about the risk of other herpes virus infections.

HSV-1 and HSV-2 and varicella zoster virus belong to the *Alphaherpesvirinae* subfamily of herpesviruses [13]. The major protective immune responses against those viruses include quick release of antiviral cytokines, recruitment and activation of natural killer (NK)-cells and CD8^+^ T cells [14]. Tofacitinib is known to inhibit signaling of antiviral cytokines such as IFNs, IL-2, and IL-4. Also, the function of NK cells and CD8^+^ T cells is inhibited by tofacitinib [7–9]. Importantly, tofacitinib is known to cross the blood-brain barrier [15]. We hypothesize that host defense against HSV-1 is dampened in the patients treated with tofacitinib and those patients have higher risk to develop encephalitis. To test our hypothesis, we have examined the effect of tofacitinib on both primary and latent herpes infection of the central nervous system (CNS) using a well-established murine model.

## METHODS

### Virus

HSV-1 (strain ID 2762) isolated from a patient with HSE (kindly provided by Professor Thomas Bergström, Department of Virology, University of Gothenburg) was grown and titrated in Vero cells.

### Mice and Infection

Male C57BL/6 mice, 6–12 weeks old, purchased from Charles River Laboratories, were housed in the animal facility of the Department of Rheumatology and Inflammation Research, University of Gothenburg. Experiments were approved by the Animal Research Ethical Committee of Gothenburg, and animal experimentation guidelines were strictly followed.

Mice were anesthetized with isoflurane (Baxter), and a total dose of 1 × 10^6^ plaque forming units (PFU) of HSV-1 was given into right and left nostril. Mice were monitored and scored according to the following: 0, no signs of infection/inflammation; 1, small nasal bump, watery eyes, jumpy behavior; 2, moderate nasal bump, conjunctivitis, ruffled hair; 3, large nasal bump, hunched/lethargic, severe conjunctivitis with swelling and hair loss, ruffled hair; 4, hunchback, severe conjunctivitis, weight loss above 20%, paralysis.

To study the reactivation of latent HSV infection, mice infected with the same HSV-1 dose were followed for 30 days. On day 30 mice were treated either with tofacitinib or vehicle. The clinical signs of HSV reactivation were monitored and scored as described above. Brains and trigeminal ganglia were collected for analyses of viral titers.

### Treatment With Tofacitinib

For in vivo pretreatment studies mice were administered tofacitinib (Hölzel Diagnistika Handels GmbH) via an Alzet miniosmotic pump (Model 2002; Durect Corporation) as previously described [16]. Content was prepared for a drug delivery of 15 mg/kg/day.

### Primary Cultures

Primary mixed glial and microglial cultures were obtained as described by Draheim et al [17]. Neuronal cultures were prepared from neonatal mice digested with 1.0 mg/mL papain/Leibowitz’s L-15 (Thermo Fisher Scientific) solution at 37°C for 15 minutes. After enzymatic digestion, cells were suspended in fresh media (Neurobasal with 2% B27 supplement, 1% Glutamax, and 1% penicillin/streptomycin; ThermoFisher) and cultured for 7 days. Monocyte cultures were prepared as described by Zhang et al [18].

### Virus Titration

Total DNA was isolated from trigeminal ganglia and brains preserved in RNA fix (Eurx) using RNA/DNA Extracol kit (Eurx), according to the manufacturer’s instructions. HSV-1 was detected using an HSV-1 probe labeled with FAM (carboxyfluorescein) in quantitative polymerase chain reaction (qPCR) instrument ViiA 7 (Fast block; Applied Biosystems) with Fast Advanced Master Mix (Thermo Fisher Scientific) as described by Namvar et al [19] and Cymerys et al [20].

### Reverse Transcription Quantitative Polymerase Chain Reaction 

Total RNA was isolated from tissue homogenates using the RNA/DNA Extracol kit (Eurx), as above. ICP0, ICP27, gB, and latency-associated transcript (LAT) were measured as described by Menendez et al [21] using GoTaq SYBR PCR System (Promega). HSV-1 LAT and lytic genes were normalized to the mean cycle threshold of β-actin. Expression of IFN-α 2, IFN-α 4, IFN-γ, IL-1β, TNF-α, IL-6, CXCL1/2, CXCL9, CXCL10, and glyceraldehyde-3-phosphate dehydrogenase (GAPDH) was quantified using Taqman Gene Expression Assays and Fast Advanced Master Mix (ThermoFisher). All PCRs were carried out in qPCR instrument ViiA 7 (Fast block; Applied Biosystems).

### Flow Cytometry Analysis

Brains and trigeminal ganglia were collected into cold phosphate-buffered saline (PBS) supplemented with 2% fetal bovine serum (FBS). Tissues were pressed through a 70-µm cell strainer and washed in PBS/2% FBS. Cell suspensions were pretreated with the Fc receptors block, rat anti-CD16/32 antibody (2.4G2; BD Biosciences). The following monoclonal antibodies were used: rat anti-CD3e-FITC (145-2C11; ThermoFisher Scientific), rat anti-CD4-PE or BV421 (RM4-5; BD Biosciences), rat anti-CD8-PE or BV421 (53-6.7; BD Biosciences), rat anti-NK1.1-APC (PK136; BD Biosciences), rat anti-CD11b-PE (M1/70; BD Biosciences), rat anti-CD192-BV421 (475301; BD Biosciences), rat anti-Ly6C-APC-Cy7 (AL-21; BD Biosciences), mouse anti-IBA-1-FITC (EPR16588; Abcam), rat anti-CD86-PE (GL1; BD Biosciences), rat anti-CD206-APC (clone MMR; ThermoFisher Scientific), rat anti-NOS2-APC (CXNFT; ThermoFisher Scientific), and rat anti-Arg-1-PE (A1exF5; eBioscience). HSV-1–specific T cells were detected with SSIEFARL-PE tetramer (ProImmune). For intracellular staining, BD cytofix/cytoperm fixation/permeabilization kit (BD Biosciences) was used according to manufacturer’s instructions. Stained cells were acquired using BD FacsLyric (BD Biosciences) and analyzed using FlowJo software (Tree Star).

### Confocal Microscopy Analysis

Cryosections were incubated overnight at 4°C with primary antibodies diluted in working solution (2% bovine serum albumin, 0.1% saponin in PBS). Antibodies used included: rabbit anti-HSV-1/2 (Dako, Agilent), APC-conjugated rat anti-CD11b (M1/70) and goat anti-IBA1 (ThermoFisher). Alexa Fluor 647-conjugated anti-goat or Alexa Fluor 488-conjugated anti-rabbit polyclonal antibodies were used for the second step (ThermoFisher). After staining with 4-6-diamidino-2-phenylindole (DAPI; ThermoFisher Scientific), sections were visualized using a Zeiss Laser Scanning Inverted Microscope LSM-700 equipped with 40X/1.3 Oil NA objective and Black Zen software (Carl Zeiss).

### Statistics

For statistical analysis, GraphPad Prism version 7 were used. To compare the differences between the groups, the Mann-Whitney *U* test and Wilcoxon test, were used and the results are reported as mean ± standard error of the mean (SEM) unless indicated otherwise. *P* < .05 was considered statistically significant.

## RESULTS

### Tofacitinib Aggravated Primary Herpesvirus Infection in Mice

**Figure 1.**
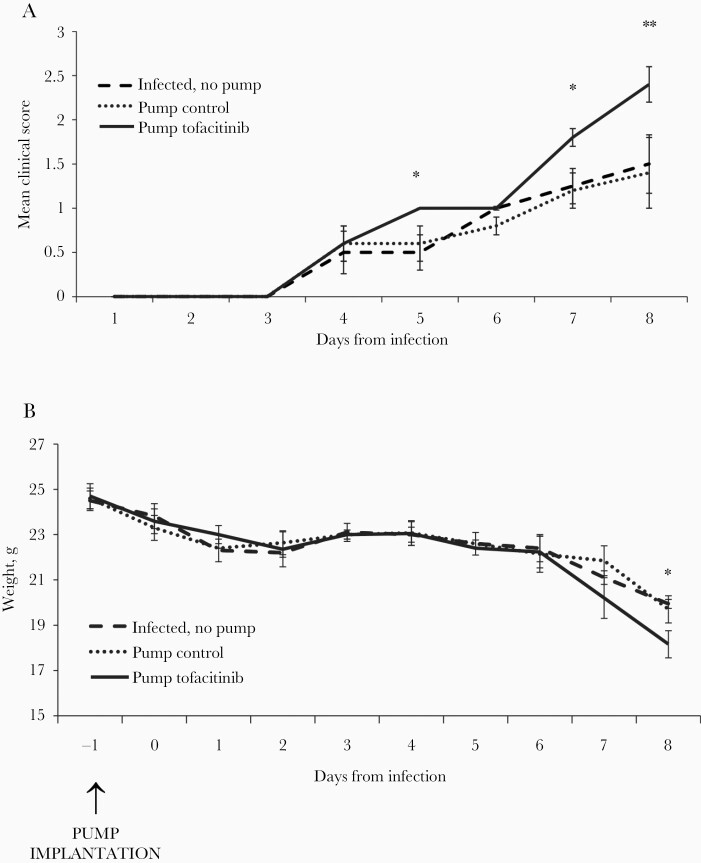
Tofacitinib aggravates primary herpesvirus infection in mice. C57BL/6 mice with or without implanted pumps releasing tofacitinib or control vehicle were intranasally infected with herpes simplex virus type 1 (HSV-1) and monitored for encephalitis symptoms (*A*) and weight change (*B*) on a daily basis. All data are presented as mean ± SEM, n = 20. Data analysis was performed by comparing animals with pumps releasing tofacitinib to animals with control pumps (containing only vehicle). ** *P* ≤ .001, * *P* ≤ .05.

To investigate whether tofacitinib impacts the course of neuroinfection by HSV-1 in C57BL/6 mice, mice pretreated for 48 hours with the drug or vehicle only, or without any pretreatment (no pump implantation) were intranasally infected with a neurovirulent strain of HSV-1. From day 5 postinfection mice treated with tofacitinib showed more severe neuroinfection compared to control mice (Figure 1A). Also, tofacitinib treatment led to significantly more weight loss compared with control groups (Figure 1B). Mice at 30 days from infection were also treated with tofacitinib for 10 days but no significant clinical differences were observed (data not shown).

### HSV-1-Infected Mice Treated With Tofacitinib Had Significantly Increased Virus Load in Both Brain and Trigeminal Ganglia

**Figure 2.**
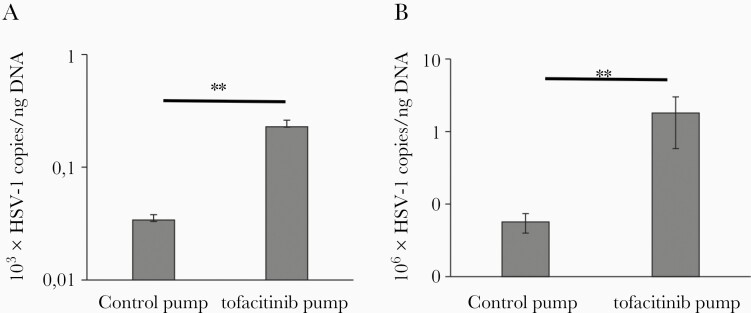
Tofacitinib increases viral loads during primary herpesvirus infection in mice. Viral loads were quantified using quantitative polymerase chain reaction (qPCR) detecting gB gene in DNA extracted from trigeminal ganglia (*A*) and brains (*B*) at 8 days postinfection in mice with implanted pumps releasing tofacitinib or control vehicle. Data are presented as mean ± SEM, n = 15. **P ≤ .001.

Trigeminal ganglia and brains isolated at day 8 were analyzed for virus titers by measuring gB copies with qPCR (Figure 2).  Tofacitinib-treated mice showed significantly higher HSV-1 titers compared to control mice (*P* ≤ .01; Figure 2). Interestingly, latently infected mice at 9 days of tofacitinib treatment showed significantly increased HSV-1 titers both in trigeminal ganglia and brains (Supplementary Figure 1A). To determine whether tofacitinib influences expression of lytic/latency genes, expression of ICP0, ICP27, and gB, indicative of an active replication, and LAT were measured in trigeminal ganglia and brains (Supplementary Figure 1B). There was a significant increase of ICP27, gB, and LAT expression in brains isolated from tofacitinib-treated mice compared to control mice (*P* ≤ .01; Supplementary Figure 1B), indicating an active replication cycle in tofacitinib-treated mice. To further clarify whether tofacitinib directly impacts replication of HSV-1 in neuronal tissue, we treated primary cell cultures of neurons, glial, and microglial cells with tofacitinib in the range of 1–300 μM. Tofacitinib had no significant influence upon replication of HSV-1, except for microglia treated with 1 μM of tofacitinib (*P* < .05; Supplementary Figure 1B).

### Effect of Tofacitinib on Production of Antiviral and Inflammatory Cytokines and Chemokines

**Figure 3.**
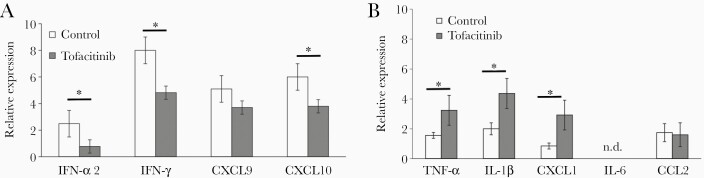
Tofacitinib influences expression of antiviral and proinflammatory cytokines and chemokines in HSV-1 infection: (*A*) antiviral cytokines (IFN-α and IFN-γ) and chemokines (CXCL9 and CXCL10); and (*B*) proinflammatory cytokines (IL-1β, TNF-α, and IL-6) and chemokines (CCL-2 and CXCL1) in the HSV-1–infected brain tissue isolated at 8 days postinfection from mice with implanted pumps releasing tofacitinib or control vehicle. Data are presented as mean ± SEM, n = 10. Data analysis was performed by comparing mice tofacitinib treated and untreated. * *P* ≤ .05. Abbreviations: CCL, chemokine ligand; CXCL, C-X-C motif chemokine ligand; HSV-1, herpes simplex virus type 1; IFN, interferon; IL, interleukin; TNF, tumor necrosis factor.

To further investigate how tofacitinib regulates antiviral and inflammatory response, expression of cytokines and chemokines was measured in brains and trigeminal ganglia isolated at day 8 of infection by qPCR (Figure 3A and 3B). The most predominant inhibition exerted by tofacitinib was observed in IFN-γ, IFN- α2, and CXCL10 (*P* < .05; Figure 3A). On the other hand, tofacitinib treatment resulted in a significant increase in TNF-α, IL-1β, and CXCL1 (*P* ≤ .05; Figure 3B). Our data demonstrate that while expression of cytokines important for anti–HSV-1 response was downregulated by tofacitinib, expression of proinflammatory cytokines and CXCL1 chemokine was upregulated.

### Tofacitinib Promotes M1 to M2 Transition of Microglia and Infiltrating Monocytes

**Figure 4.**
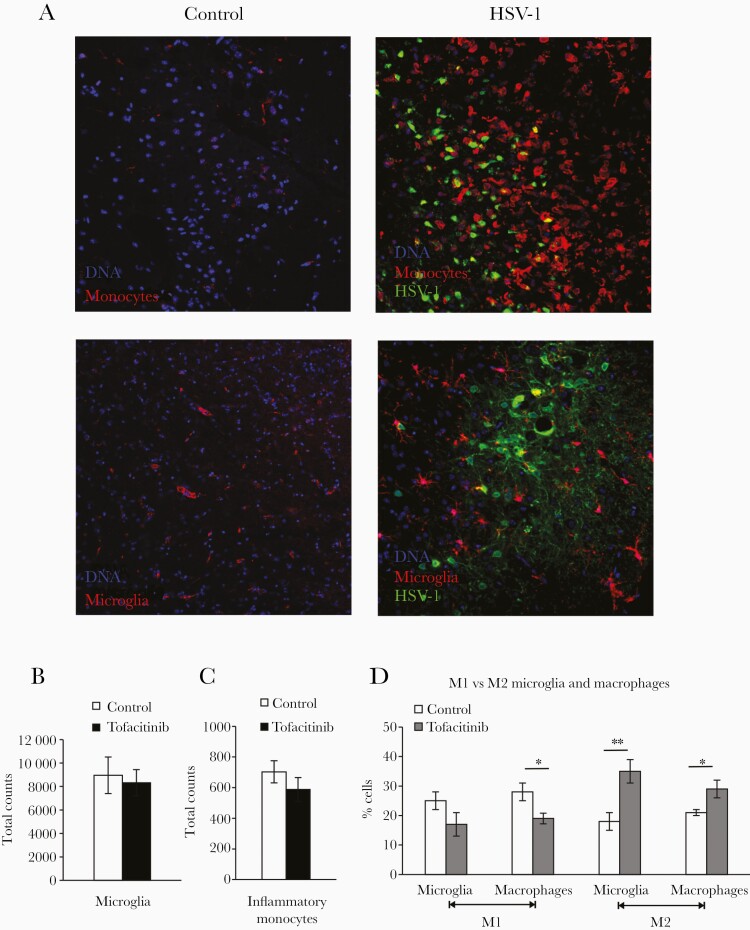
Tofacitinib promotes M1 to M2 transition of microglia and infiltrating monocytes. *A*, Representative confocal microphotographs of infiltrating monocytes (upper panel) and microglia (lower panel) in uninfected and herpes simplex virus type 1 (HSV-1)–infected midbrains at 8 days postinfection. Coimmunefluorescent staining for HSV-1 antigens (green), CD11b^+^ monocytes (red), and IBA-1 positive microglia (red). Nuclei (blue) were counterstained with 4-6-diamidino-2-phenylindole (DAPI). Magnification × 200. *B*, Microglia cell counts, (*C*) monocyte cell counts, and (*D*) M1/M2 phenotype of microglia and infiltrating monocytes measured by flow cytometry in the homogenates of HSV-1–infected brain tissue isolated at 8 days postinfection from mice with implanted pumps releasing tofacitinib or control vehicle. Data are presented as mean ± SEM, n = 7. **P ≤ .001, *P ≤ .05.

Resident microglia and infiltrating monocytes are 2 main myeloid cells of the CNS, which contribute to brain inflammation in HSV-1 infection [22]. In mice with HSV-1 encephalitis, infiltrating monocytes accumulate around HSV-1–infected cells in brains, and a similar effect but with less extent was also observed for microglia (Figure 4A). The total numbers of both microglia (Figure 4B) and infiltrating monocytes (Figure 4C) in brains were similar at day 8 postinfection in both groups. When we subgrouped the cells to M1 and M2 cell populations, a decrease in the percentage of M1 phenotype in tofacitinib-treated mice was observed for monocytes (*P* < .05; Figure 4D). In contrast, the percentages of M2 phenotype in microglia and monocytes from tofacitinib-treated mice were significantly higher (*P* ≤ .05; Figure 4D). Our data suggest that tofacitinib promotes the M1 to M2 transition for both microglia and infiltrating monocytes. Flow cytometry analysis of CD4^+^ T cells, CD8^+^ T cells, and NK cells indicated that tofacitinib treatment leads to significantly increased infiltration of cytotoxic T cells (CD8^+^) and HSV-1–specific cytotoxic T cells (CD8^+^/SSIEFARL^+^) (*P* ≤ .05), while tofacitinib does not affect numbers of helper CD4^+^ T cells and NK cells in brain (Supplementary Figure 2).

### Tofacitinib Inhibited Macrophage Activation In Vitro and Increased HSV 1 Titers

To study how HSV-1 infection influences the phenotype of myeloid cells, we used in vitro models of primary microglia and peritoneal macrophages. After 24 hours of HSV-1 infection, primary microglia and macrophage cultures showed a significantly decreased M1 and increased M2 phenotype in comparison to uninfected controls (*P* ≤ .01; Supplementary Figure 3), suggesting that HSV-1 infection itself induces M1 to M2 transition.

**Figure 5.**
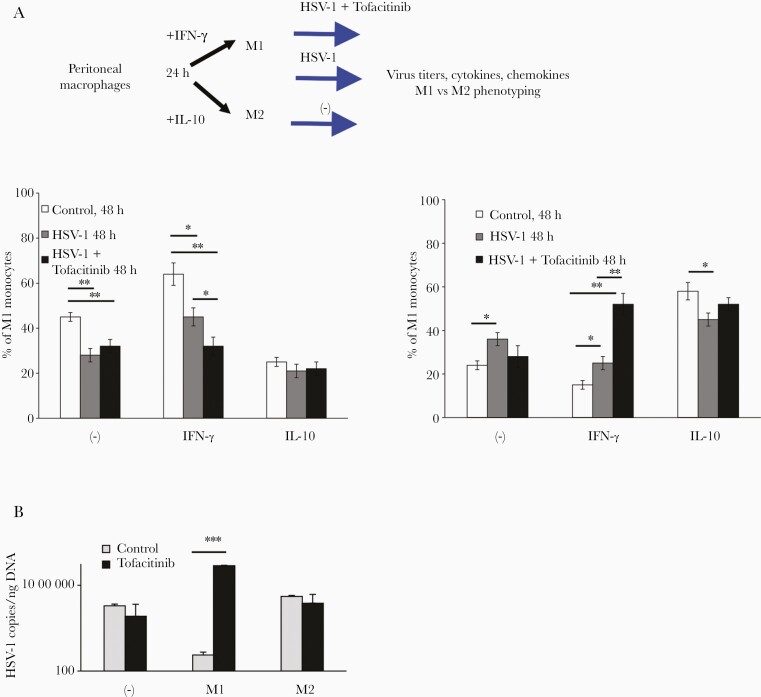
Tofacitinib inhibits macrophage activation in vitro and increases HSV-1 titers. Peritoneal macrophages isolated from C57BL/6 mice were differentiated into M1 and M2 phenotype, infected with HSV-1, and treated or not with tofacitinib, and further analyzed for M1/M2 phenotypes (*A*) and HSV-1 titers at 24 hours postinfection (*B*). Data are presented as mean ± SEM, n = 3. ***P ≤ .001, **P ≤ .01, *P ≤ .05.

To further understand how tofacitinib influences macrophage activation during HSV-1 infection, we differentiated macrophages into M1 and M2 phenotype and infected the cells in the presence of tofacitinib (Figure 5A). HSV-1–infected M1 macrophages treated with tofacitinib showed significantly lower percentage of M1 phenotype in comparison to HSV-1–infected cultures without tofacitinib treatment (*P* < .05; Figure 5A). On the contrary, M1-differentiated cultures showed a significantly higher percentage of M2 phenotype with tofacitinib present at infection (*P* < .01; Figure 5A). Tofacitinib did not influence the phenotype of already differentiated M2 cells (Figure 5A). Interestingly, tofacitinib significantly increased the numbers of HSV-1 copies in M1 cultures, in comparison to nontreated infected M1 macrophages (*P* < .05; Figure 5B).

### Tofacitinib Inhibited Production of Inflammatory and Antiviral Cytokines by Macrophages In Vitro

We next analyzed how tofacitinib influences production of antiviral (IFN-α4, CXCL9, and CXCL10) and inflammatory (CXCL1/2, IL-1β, TNF-α, and IL-6) cytokines and chemokines by M1/M2 macrophages (Supplementary Figure 5). HSV-1 infection upregulated IFN-α4, CXCL10, IL-6, IL-1β, and TNF-α expression in macrophage cultures (*P* ≤ .05; Supplementary Figure 4). HSV-1–infected M1 macrophages upregulated gene expression of CXCL9, CXCL10, and TNF-α (*P* ≤ .05; Supplementary Figure 4), while infected M2 macrophages produced significant amounts of IL-6, IL-1β, and CXCL1/2 (*P* ≤ .05; Supplementary Figure 4). Notably, tofacitinib blocked upregulation of IFN-α4 CXCL9, and CXCL10 mRNAs as well as of IL-6, IL-1β, and TNF-α mRNAs in control and HSV-1–infected cultures, irrespectively of the phenotype (*P* ≤ .05; Supplementary Figure 4). The only exception was detected for CXCL1 chemokine, for which tofacitinib upregulated expression in both control undifferentiated, M1/2 macrophages as well as for HSV-1–infected undifferentiated and M2 macrophages (*P* ≤ .05; Supplementary Figure 4). To further check how tofacitinib influences expression of antiviral IFN-α4, CXCL9, and CXCL10 in microglia, we measured mRNAs by qPCR in primary microglia and mixed glial cultures (Supplementary Figure 5). While poly I:C significantly increased expression of IFN-α4, CXCL9, and CXCL10 mRNAs by both mixed glial and microglia cultures, addition of tofacitinib totally inhibited the observed effect (*P* ≤ .001 for CXCL9 and CXCL10). Tofacitinib also significantly downregulated expression of CXCL9 and CXCL10 gene expression induced by HSV-1 (*P* ≤ .05). However, tofacitinib did not influence IFN-α4 mRNAs in HSV-1–infected microglia and mixed glial cultures (Supplementary Figure 5).

## DISCUSSION

Our data demonstrate that tofacitinib treatment in HSV-1 primary infection worsened clinical outcome and increased viral load in CNS by hampering the antiviral response induced by monocytes and microglia. In contrast, tofacitinib treatment did not cause any clinical reactivation of latent HSV-1 despite significantly higher virus loads found in CNS compared to the controls. It has been shown that reactivation of latent, 30-day HSV-1 infection in mice by hypothermia leads to reactivation of the virus in brains but not in trigeminal ganglia [23]. However, multiple reactivation of latent infection leads to neuroinflammation within CNS, followed by cognitive deficits [24].

While tofacitinib had no direct effect upon replication of HSV-1, it led to a decreased production of antiviral cytokines and chemokines involved in anti–HSV-1 response, such as IFN-α, IFN-γ, and CXCL9/10. Type I IFN subtypes (α/β) are considered indispensable for antiviral protection while IFN-γ, a type II IFN, is essential for clearing brain of HSV infection [25]. CXCL9 and CXCL10 are expressed at high levels in HSV-1–infected brains, primarily in microglia and infiltrating monocytes and are necessary for recruitment of natural killer cells and CD8^+^ T-cells [26, 27].

Infiltrating monocytes play a central role in acute and chronic HSV-1 infection, as they can act to either exacerbate or protect against infection [26, 27]. Macrophages can differentiate into proinflammatory M1 macrophage, or anti-inflammatory M2 macrophage subtypes [28]. M1 macrophages are induced by IFN-γ and produce toxic effector molecules such as reactive oxygen species and nitric monoxide (NO), as well as inflammatory cytokines such as IL-1β, TNF, and IL-6 [28, 29]. In contrast, M2 macrophages produce high levels of anti-inflammatory cytokines such as IL-10 and transforming growth factor β [28, 29]. M1 macrophages are refractory to HSV-1 replication in vitro compared to M2 or unpolarized macrophages [30]. Jaggi et al showed that that overexpression of M2 macrophages in HSV-1 infection results in increased viral replication and viral load, as well as increased expression of both pro- and anti-inflammatory cytokines [31]. Here, we found that while tofacitinib treatment did not significantly influence infiltration of monocytes during HSV-1 infection, it shifted macrophages to M2 phenotype both in vitro and in vivo. Furthermore, tofacitinib facilitated HSV-1 replication in M1 infected macrophages, most probably by stabilizing HSV-1–induced transition to M2 phenotype and consequently less NO production.

In our study, addition of tofacitinib downregulated expression of antiviral IFN-α4, CXCL9, and CXCL10, as well as proinflammatory IL-6, IL-1β, and TNF, by unpolarized, but also M1/M2 polarized, HSV-1–infected and uninfected macrophages. This is in line with previous report showing decreased expression of CXCL10, TNF, and IFN-α by tofacitinib-treated murine macrophages [32, 33]. Interestingly, tofacitinib induces expression of CXCL1 chemokine, which acts as a chemoattractant for several immune cells and plays an important role in regulation of immune and inflammatory responses [32, 33]. Microglial cells are crucial in maintaining brain homeostasis and protection of the brain against pathogens [34]. The role of microglia in the context of HSV-1 infection of the CNS remains poorly explored. In this study, tofacitinib treatment of mice led to M2 microglia polarization. We speculate that this polarization may also hamper anti–HSV-1 immune response in CNS, as early microglial response followed by sustained infiltration of monocytes and T cells into the brain seem to be key components for a better clinical outcome in mouse model of HSE [29].

Surprisingly, while tofacitinib decreased production of antiviral chemoattractants, we noted significantly increased infiltration of cytotoxic T cells, in particular HSV-1–specific CD8^+^ T cells. As the specimens were collected at a relatively late stage of the disease (day 8 after infection), we speculate that the increased infiltrating of T cells simply reflects the severity of infections and higher virus load in brain tissues treated with tofacitinib. This is actually in line with the finding that tofacitinib treatment also led to significant upregulation of proinflammatory cytokines, such as IL-1β, TNF-α, and CXCL1/2 chemokine, although tofacitinib is known to block TNF-α and IL-1β production [32]. However, while T-cell numbers were high, tofacitinib may inhibit their ability to clear virus from the brain tissue. It is well known that inflammatory response that develops during HSV-1 infection of CNS is a “double-edged sword” as it is critical to control viral replication in the brain early after infection, but it may also result in an exaggerated neuroinflammation leading to poor clinical outcome [2, 27].

What is the clinical relevance of our findings? As most people were already exposed to herpes at a young age [1], the risks associated with primary herpes infection are obviously not the major concerns in the adult patients receiving tofacitinib. For those patients, whether tofacitinib treatment reactivates herpes simplex is a more clinically relevant question. JAK inhibitors in clinical practice are used for prolonged periods (months to years). In the current study, the duration of tofacitinib treatment was limited to 10 days due to practical issues. It is possible that short-term treatment is insufficient to induce clinical encephalitis. Therefore, the effect of prolonged tofacitinib treatment in latent HSV needs to be further studied. There are substantial numbers of primary herpes infections in the pediatric patient group. Recently, tofacitinib was approved for treatment of juvenile arthritis from the age of 2 years [35]. Introducing a potentially increased risk of a serious complication such as encephalitis, where early treatment is of the essence, is vital information for patients. Our results highlight a potential elevated risk of disease aggravation and development of severe encephalitis in primary herpes infection by tofacitinib treatment. Children receiving tofacitinib treatment might benefit from this information and increased awareness about possible consequence of primary herpes infection. Importantly, physicians may need to treat those patients with antiviral drugs more liberally. Further safety studies in this population could benefit from our findings and will give us more detailed information about the clinical relevance of these findings.

## Supplementary Data

Supplementary materials are available at *The Journal of Infectious Diseases* online. Supplementary materials consist of data provided by the author that are published to benefit the reader. The posted materials are not copyedited. The contents of all supplementary data are the sole responsibility of the authors. Questions or messages regarding errors should be addressed to the author.

jiac040_suppl_Supplementary_Figure_S1

jiac040_suppl_Supplementary_Figure_S2

jiac040_suppl_Supplementary_Figure_S3

jiac040_suppl_Supplementary_Figure_S4

jiac040_suppl_Supplementary_Figure_S5

## Notes

***Financial support.*** This work was supported by Vastra Götaland Regionen (More2020 scholarship to M. K.); Rune and Ulla Amlövs Stiftelse (to K. E., T. J., and A. J.); Wenner-Gren Foundation (grant number GFoh2019-0012 scholarship to M. K.); Swedish Medical Research Council (grant numbers 523-2013-2750 and 2019-01135 to T. J., and 2020-02732 to K. E.); the Swedish Government under the ALF agreement with county councils (grant numbers ALFGBG-823941 and ALFGBG-933787 to T. J., ALFGBG-770411 to A. J., and ALFBGB-827291 to K. E.); and Sahlgrenska University Hospital Foundations (to A. J.).

***Potential conflicts of interest.*** All authors: No reported conflicts of interest. All authors have submitted the ICMJE Form for Disclosure of Potential Conflicts of Interest. Conflicts that the editors consider relevant to the content of the manuscript have been disclosed.
