# Supplementary figures and images for: Tofacitinib Treatment in Primary Herpes Simplex Encephalitis Interferes With Antiviral Response
Source: J Infect Dis. 2022 Feb 26;225(9):1545–53. doi: 10.1093/infdis/jiac040 (PMC9635063; doi:10.1093/infdis/jiac040)

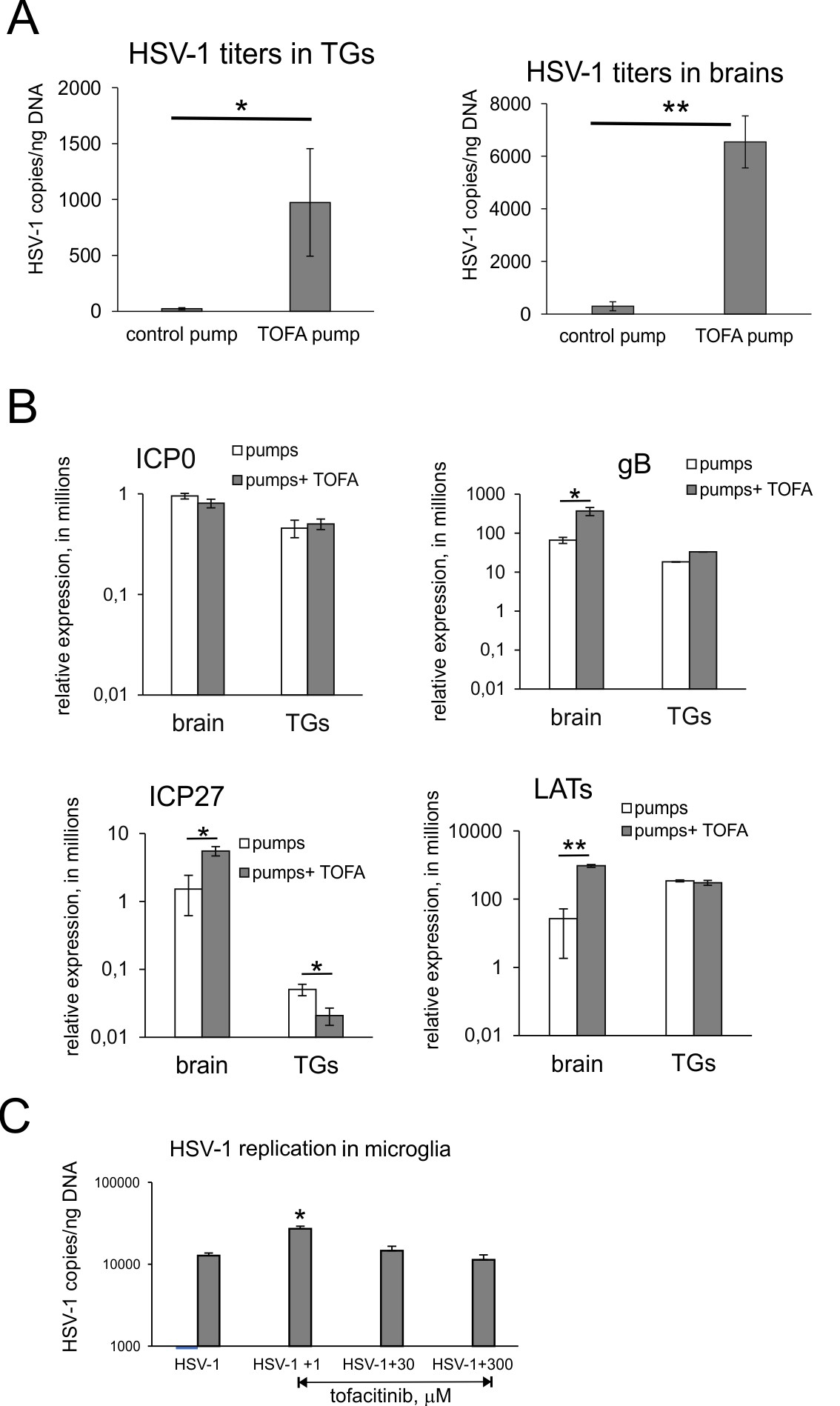

Supplement: jiac040_suppl_Supplementary_Figure_S1 [file jiac040_suppl_supplementary_figure_s1.jpeg]

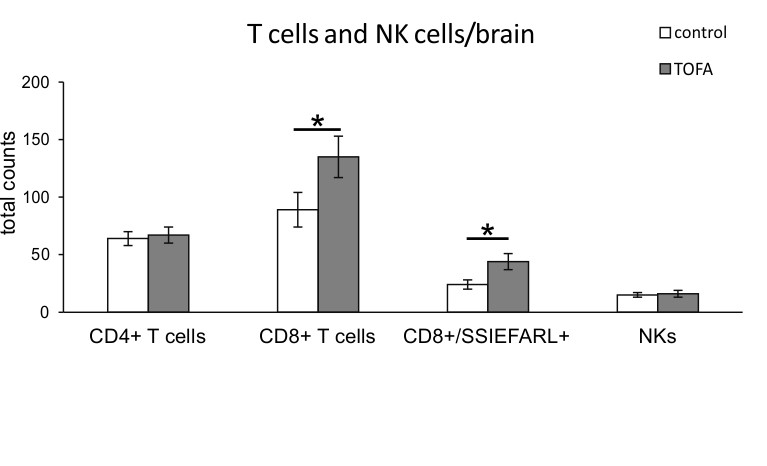

Supplement: jiac040_suppl_Supplementary_Figure_S2 [file jiac040_suppl_supplementary_figure_s2.jpeg]

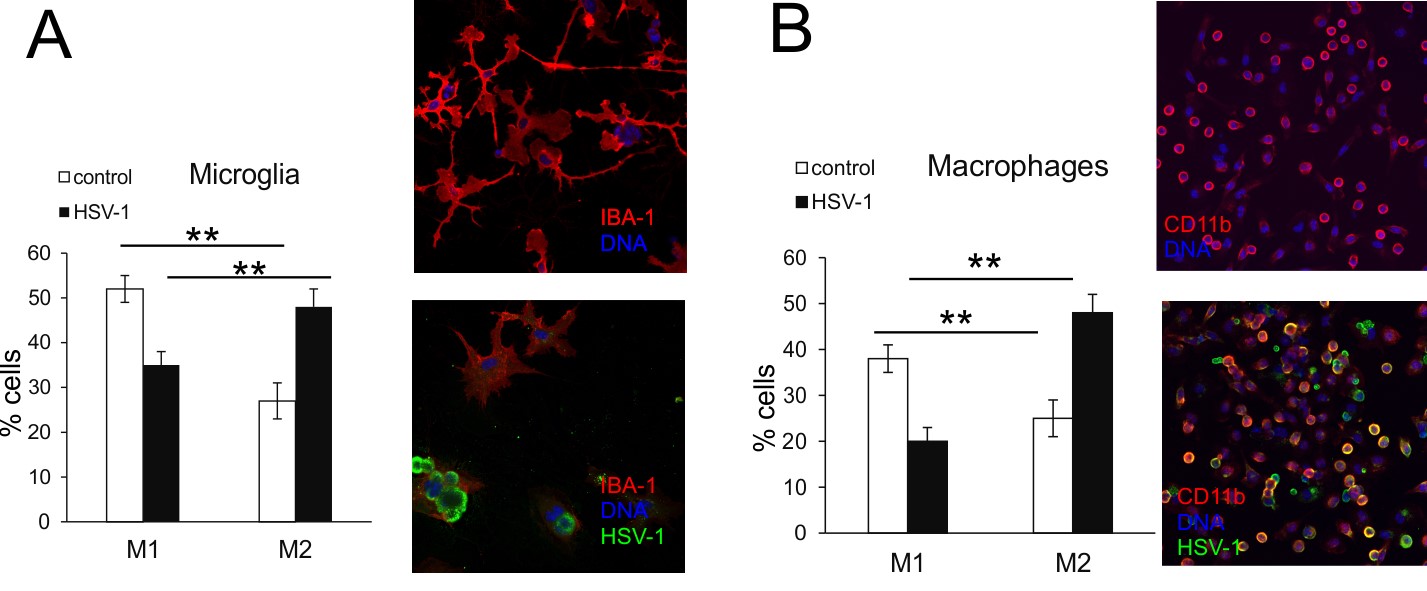

Supplement: jiac040_suppl_Supplementary_Figure_S3 [file jiac040_suppl_supplementary_figure_s3.jpeg]

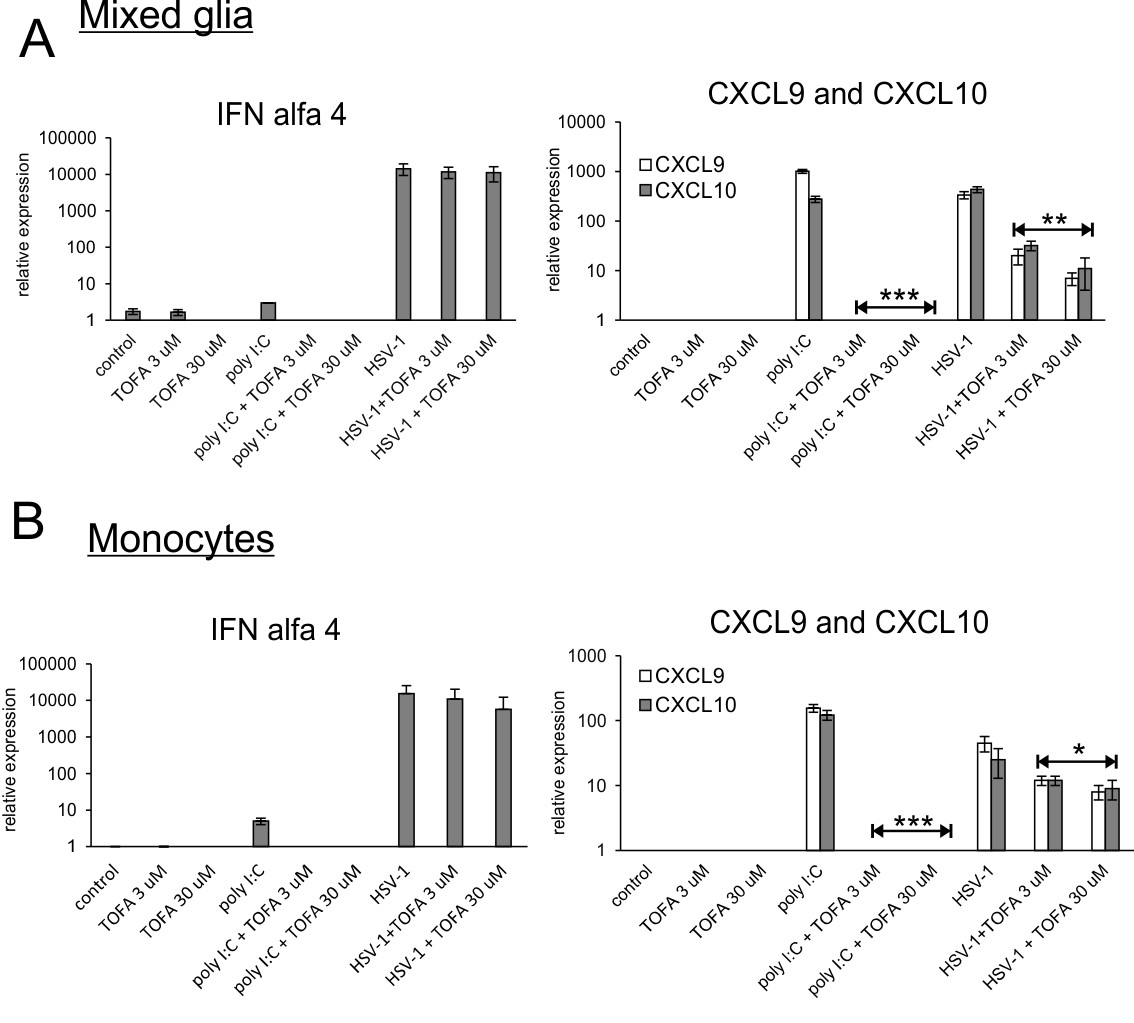

Supplement: jiac040_suppl_Supplementary_Figure_S4 [file jiac040_suppl_supplementary_figure_s4.jpeg]

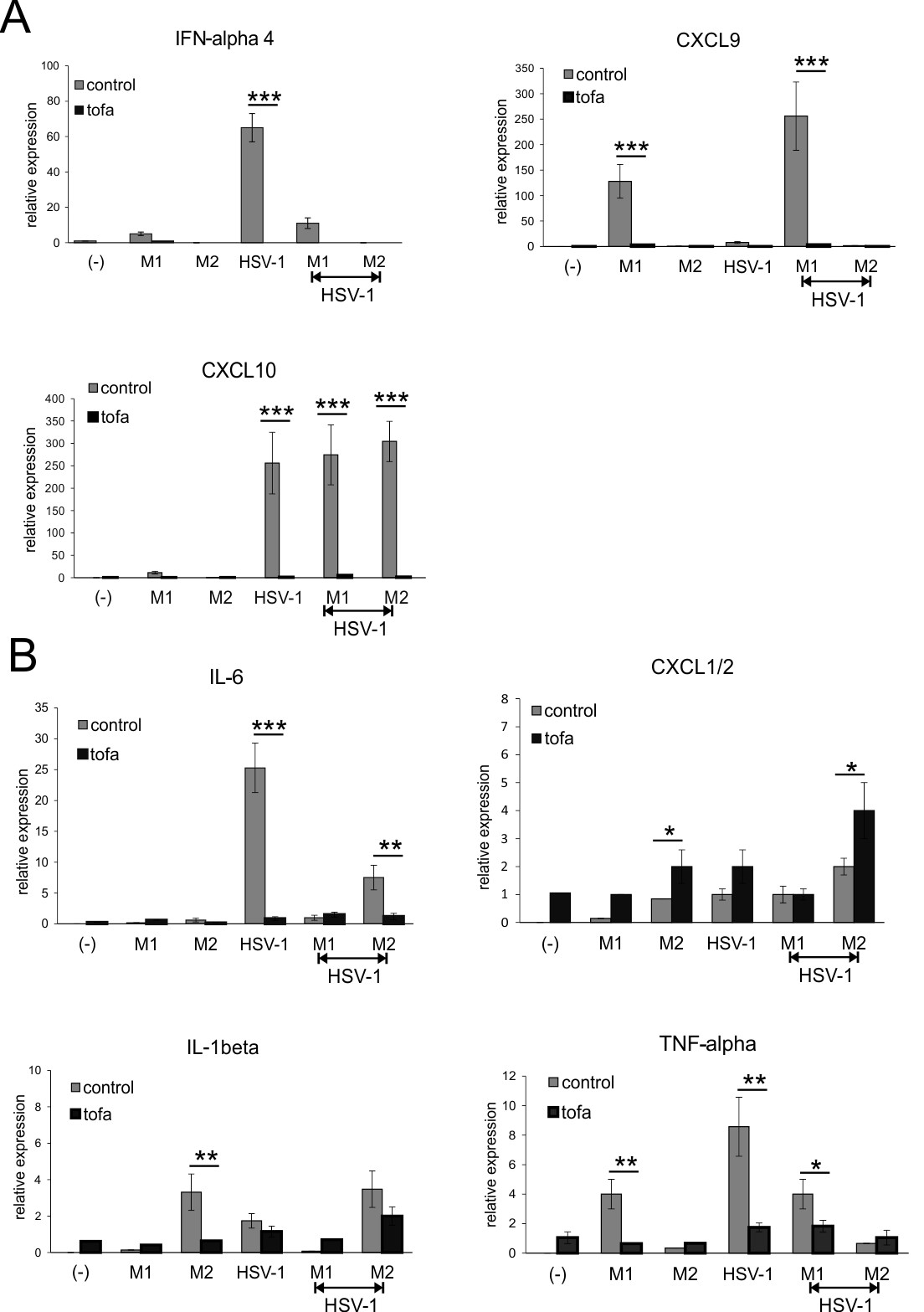

Supplement: jiac040_suppl_Supplementary_Figure_S5 [file jiac040_suppl_supplementary_figure_s5.jpeg]
